# Dr. Burroughs and His Borrowed Discovery

**Published:** 1889-10

**Authors:** 


### EDITORIALS AND MISCELLANEOUS.

### DR. BURROUGHS AND HIS BORROWED DISCOVERY.

The medical literature of the day is teeming with quotations from and comments on the article published lately by Dr. Burroughs in the *London Lancet*, in which he claims to have made some wonderful experiments and startling discoveries in the use of nitro-glycerine as a therapeutic agent.

In the September issue, 1887, of the SOUTHERN MEDICAL RECORD, that is two years ago, we published an article from the pen of Dr. E. Van Goidtsnoven, of Atlanta, Georgia, giving the same series of experiments as claimed by Dr. Burroughs, and detailing the same results. In proof thereof we are prepared to send a copy of The RECORD containing the original article, ideas and propositions to those who may wish to inquire into the true merits of the case. By comparing the two productions and their respective dates of issue, it can be easily ascertained who purloined the other's ideas and published them as his own.

Dr. Van Goidtsnoven is one of the collaborators of the SOUTHERN MEDICAL RECORD and it is but justice to him and to our Journal that the matter should be set aright.

In connection with the above we publish the following correspondence kindly furnished us by Dr. Van Goidtsnoven:

Popular Science News and Boston Journal of Chemistry,
Haverhill, Mass., Sept. 12, 1889.

Dr. E. Van Goidtsnoven,

Dear Sir:—I have your card of the 6th, and copy of the SOUTHERN MEDICAL RECORD, which evidently proves that you have anticipated Dr. Burroughs by a considerable length of time. I think I have also seen mention made of these properties of nitro-glycerine in other medical journals, bnt am not quite sure whether you claim the discovery as original with you or not.

Yours truly,
AUSTIN P. NICHOLS.

Atlanta, Ga., Sept. 16th, 1889.

Mr. Austin P. Nichols, S. B. Haverhill, Mass.,

My Dear Sir:—With many thanks I acknowledge the receipt of your kind favor of the 12th instant.

I lay no claim to the discovery of nitro-glycerine as a therapeutic agent, and the prefatory remarks that are published in my article on "*dynamite*" sufficiently grant that.

Nitro-glycerine was discovered by Sobrero in 1847 and has been used ever since in the treatment of nephritis and angina pectoris, especially by the Homœopathic School under the mask of glonoin. I simply claim that I originated a series of experiments which in my humble opinion greatly widen the usefulness of nitro-glycerine as a therapeutic agent In proof whereof I respectfully refer you to Sajous Annual of the Universal Medical Sciences, issue of 1888, volume IV., page 488.

I further claim that no disinterested party can read my report and that of Dr. Burroughs without being forcibly impressed with the conviction that the

*production* published in the *London Lancet*, June, 1889 is but the *reproduction* of what was published in the SOUTHERN MEDICAL RECORD September, 1887, by a different author. I am, my dear Sir, Yours very respectfully,

E. VAN GOIDTSNOVEN, M. D.

